# Prevalence and Correlates of Metabolic Syndrome in Chinese Children: The China Health and Nutrition Survey

**DOI:** 10.3390/nu9010079

**Published:** 2017-01-18

**Authors:** Peige Song, Jinyue Yu, Xinlei Chang, Manli Wang, Lin An

**Affiliations:** 1 Department of Child, Adolescent and Women’s Health, School of Public Health, Peking University, Beijing 100191, China; peigesong@hsc.pku.edu.cn (P.S.); changxl_echo@163.com (X.C.); 18354226871@163.com (M.W.); 2 Centre for Global Health Research, Usher Institute of Population Health Sciences and Informatics, University of Edinburgh, Edinburgh EH8 9AG, UK; 3 Division of Medicine, School of Life and Medical Science, University College London, London WC1E 6BT, UK; rmhajyy@ucl.ac.uk

**Keywords:** children, metabolic syndrome, epidemiology, China

## Abstract

Metabolic syndrome (MetS) is generally defined as a cluster of metabolically related cardiovascular risk factors which are often associated with the condition of insulin resistance, elevated blood pressure, and abdominal obesity. During the past decades, MetS has become a major public health issue worldwide in both adults and children. In this study, data from the China Health and Nutrition Surveys (CHNS) was used to assess the prevalence of MetS based on both the National Cholesterol Education Program Adult Treatment Panel III (NCEP-ATPIII) guidelines and the International Diabetes Federation (IDF) criteria, and to evaluate its possible correlates. A total of 831 children aged 7–18 years were included in this study, and 28 children were classified as having MetS as defined by the modified NCEP-ATPIII definition, which yielded an overall prevalence of 3.37%. Elevated blood pressure was the most frequent MetS component. The results of logistic regression models revealed that increased body mass index (BMI), hyperuricemia, and insulin resistance (IR) were all associated with the presence of MetS. To conclude, our study revealed the prevalence of MetS in Chinese children at the national level. Further large-scale studies are still needed to identify better MetS criteria in the general paediatric population in China.

## 1. Introduction

Metabolic syndrome (MetS) is generally defined as a cluster of metabolically related cardiovascular risk factors which are often associated with the condition of insulin resistance, elevated blood pressure, and abdominal obesity [1]. Clinical research indicates that the MetS patients are at high increased risk for the incidence of cardiovascular diseases, type II diabetes mellitus, and all-cause mortality [2]. In adults, the definition of MetS varies in terms of the indicators and the corresponding cut-offs. The most commonly used criteria for MetS diagnosis in recent research includes the World Health Organization (WHO) criteria [3], the National Cholesterol Education Program Adult Treatment Panel III (NCEP-ATPIII) guidelines [4,5,6], the International Diabetes Federation (IDF) criteria [7,8], and the statement from the American Heart Association (AHA) and the National Heart, Lung, and Blood Institute (NHLBI) in 2005 [9].

During the past decades, MetS has becoming a major public health issue worldwide [10]. Recent data indicates that the global prevalence of MetS ranges from <10% to as much as 84%, based on different diagnostic criteria [1]. From 2000 to 2005, the overall estimates for the MetS prevalence based on the NCEP-ATPIII guidelines increased from 22% to 34% among USA adults [11,12]. In China, the observed MetS prevalence was 21.3% and 18.2%, according to the modified NCEP-ATPIII and IDF criteria, respectively [13]. Epidemiological studies show that higher socioeconomic status, sedentary lifestyle, central obesity, and high waist circumference were significantly associated with the development of MetS [1,14]. 

The indicators of MetS are also present in early life, and paediatric obesity has been considered as one of the most important predictors for the development of MetS [1,15]. Due to the rapid development of biological characteristics in children, there is a lack of universally accepted definitions for MetS in this younger age group, and most studies adopt or modify the criteria of NCEP-ATP III and IDF [6,8].

In China, the nutritional transition has made the epidemiology of paediatric obesity and related chronic conditions into public concerns in the past decades [16,17]. The increased prevalence of overweight and obesity rates among children has been observed in a number of studies [16,18]. A national survey reported that the prevalence of paediatric obesity has increased by almost five times from 1985 to 2000; in 2010 the observed prevalence of overweight and obesity among children had reached 9.9% and 5.5%, respectively [18]. With the increased rate of paediatric obesity in Chinese children, it is also likely that the prevalence of MetS has increased in Chinese children. Although MetS has been studied extensively in adults, there is still a lack of studies on characterising MetS in children and adolescents in terms of criteria, prevalence, or clinical implications at the national level [16,19].

In this study, data from the China Health and Nutrition Surveys (CHNS) [20,21]—a national population-based study—was used to assess the prevalence of MetS according to both the NCEP-ATPIII and IDF definitions in Chinese children and to evaluate its possible correlates.

## 2. Materials and Methods

### 2.1. Study Design and Participants

CHNS is an ongoing national household-based study which adopted a multistage random cluster sampling method in nine provinces (Guangxi, Guizhou, Heilongjiang, Henan, Hubei, Hunan, Jiangsu, Liaoning, and Shandong) with different geographies, economic development levels, and health indicators in China [20,21]. CHNS was conducted successively in the years 1989, 1991, 1993, 1997, 2000, 2004, 2006, 2009, and 2011. At the individual level, the response rates were high for each wave of the survey from 1989 to 2006 (averaging 88%) [21]. For our purpose of assessing paediatric MetS, the availability of biomarker data is essential; thus, the CHNS 2009 data was used in our analysis, where the fasting blood data was available for children aged 7 years and above [13]. The study protocols were approved by the Institutional Review Boards of the University of North Carolina, Chapel Hill, and the Chinese Centre for Disease Control, and all children and their parents provided written informed consent [20,21].

### 2.2. Sample Size

The prevalence of MetS in Chinese children was estimated as 1.8% to 3.2% according to different definition criteria [19]. To achieve a 2.6% predicted prevalence, the minimum number of required children was 529 with 95% level of confidence and 3% bound on the error of estimation. 

### 2.3. Anthropometric and Clinical Measurements

Standard procedures were followed by well-trained examiners [22]. Weight was measured to the nearest 0.1 kg with lightweight clothing on a calibrated beam scale. Height was measured to the nearest 0.1 cm without shoes using a portable stadiometer. Body mass index (BMI) was calculated as weight in kilograms divided by the square of height in meters. Waist circumference (WC) was measured at a midpoint between the lowest rib and the iliac crest in a horizontal plane by using non-elastic tape. After a 10-min seated rest, blood pressure (BP) measurements were taken in triplicate by trained and qualified observers using a mercury sphygmomanometer according to the standard protocol [23,24], and an appropriate cuff size was used for children; Korotkoff phase 1 and Korotkoff phase 5 were defined as systolic blood pressure (SBP) and diastolic blood pressure (DBP), respectively. The mean of the three BP measurements was used in the analyses.

After at least 8 h of overnight fasting, blood samples were collected from household respondents aged 7 years and older. Then, the blood samples were tested immediately for glucose and haemoglobin A1c, and then the plasma and serum samples were frozen and stored at −86 °C for later laboratory analysis. Finally, all samples were analysed in a national central lab in the capital with strict quality control. Glucose was analysed by the glucose oxidase—phenol and aminophenazone (GOD-PAP) method, insulin by the radioimmunology method, serum uric acid (UA) by the enzymatic colorimetric method, haemoglobin (Hb) by the volume, conductivity, light scatter (VCS) method, total cholesterol (TC) by the cholesterol oxidase—phenol and aminophenazone (CHOD-PAP) method, high-density lipoprotein cholesterol (HDL) and low-density lipoprotein cholesterol (LDL) by the enzymatic method, and triglyceride (TG) by the glycerol-3-phosphate oxidase—phenol and aminophenazone (GPO-PAP) method.

### 2.4. Definitions

Overweight was defined as a BMI ≥85th percentile and <95th percentile for gender and age, and obesity was defined as a BMI greater than or equal to the gender- and age-specific 95th percentile according to the Chinese BMI classification for children [25]. No universally-accepted threshold defines hyperuricemia in children; in this study, we defined hyperuricemia using the threshold of a UA value ≥357 μmol/L in accordance with previous studies [26]. Anaemia was defined according to the WHO criteria as a Hb <115 g/L for children aged ≥5 and <12 years, <120 g/L for children aged ≥12 and <15 years, <120 g/L for girls aged ≥15 years, and <130 g/L for boys aged ≥15 years [27]. Insulin resistance (IR) is affected by age and pubertal status [28], but no Tanner stage data was available for all participants in the database. To assess the age-related associations of MetS and IR, all children were divided into three age groups (7–10, 11–13, and 14–18 for girls; 7–11, 12–14, and 15–18 for boys) to reflect the prepubertal, pubertal, and postpubertal stages, respectively, according to the Chinese classification [29,30,31]. Currently, no universal definition of IR is applicable in normal and overweight children, so we adopted the 75th percentile of the homeostasis model assessment (HOMA: fasting serum insulin (μU/mL) × fasting plasma glucose (mmol/L)/22.5) within each age group as the threshold of IR [5,32]. The IR thresholds assessed by the HOMA index are listed in Table 1.

In this study, MetS and its components in children aged 7–18 years were defined according to the modified criteria of the NCEP-ATP III [6]. MetS was identified when three or more of the following five components were present: (1) abdominal obesity: a WC equal to or above the gender- and age-specific 90th percentile for Chinese children [33]; (2) elevated TG: a TG ≥110 mg/dL; (3) low HDL: a HDL ≤40 mg/dL; (4) elevated blood pressure: an SBP and/or a DBP ≥90th percentile for gender, age, and height [24]; (5) elevated fasting glucose: a glucose ≥110 mg/dL. Moreover, the IDF definition was also applied to explore the concordance with the NCEP-ATP III definition in children aged 10–18 years [8]. 

### 2.5. Statistical Analysis

Data are reported in accordance with the Strengthening the Reporting of Observational Studies in Epidemiology (STROBE) statement [34]. Data of the basic demographic, anthropometric, and clinical parameters, and MetS components were presented for children with and without MetS. Categorical data were expressed as percentages, and continuous data were expressed as means (±SD); for insulin and HOMA-IR, data were expressed as median with lower and upper quartiles because of the skewed distribution. Comparisons were performed by *t*-test and χ^2^-test for continuous data and categorical data, respectively, and by Mann–Whitney U test for insulin and HOMA-IR. The gender-specific prevalence of MetS was calculated. Binary logistic regression models were adopted to examine the association between the presence of MetS and the associated correlates. The adjusted odds ratios (ORs) and 95% confidence intervals (CIs) were presented. In children aged 10–18 years, the agreement between different MetS definitions (NCEP-ATP III and IDF) was evaluated by the Cohen’s kappa coefficient (κ), where κ ≤ 0.20 indicates “poor”, κ = 0.21–0.40 “fair”, κ = 0.41–0.60 “moderate”, κ = 0.61–0.80 “substantial”, and κ > 0.80 “very good” [13]. All analysis was conducted in SPSS 22.0 (IBM Corporation, Armonk, NY, USA), and a two-tailed *p* value < 0.05 was considered statistically significant. 

## 3. Results

### 3.1. Basic Characteristics of the Study Subjects

A total of 831 children aged 7–18 years were included in this study, and the basic demographic, anthropometric, and clinical characteristics of the study subjects according to their MetS status are presented in Table 2. The mean age of the subjects was 12.39 ± 3.05 years. Twenty-eight subjects had MetS, whereas 803 were free of MetS. Subjects with MetS had higher values of weight, BMI, WC, SBP, DBP, UA, TG, insulin, and HOMA-IR, but lower HDL values than subjects without MetS. Seventy-five subjects had abdominal obesity, and were younger and with higher values of weight, height, BMI, WC, SBP, DBP, UA, TG, insulin, and HOMA-IR than subjects without abdominal obesity. Subjects with elevated TG (*n* = 191) were older, and had higher values of weight, height, BMI, WC, UA, TC, TG, glucose, insulin, and HOMA-IR, but lower HDL than those without elevated TG. Compared with subjects without low HDL, subjects with low HDL (*n* = 74) were older and had higher values of WC, UA, TG, but lower values of TC and HDL. Subjects with elevated blood pressure (*n* = 199) were generally younger, and had higher values of SBP, DBP, insulin, and HOMA-IR, but lower values of height than those without elevated blood pressure. For subjects with elevated fasting glucose (*n* = 14), they were found having higher values of UA, TG, glucose, insulin, and HOMA-IR than those without elevated fasting glucose. 

### 3.2. Prevalence and Distribution of MetS Components

**Figure 1:**
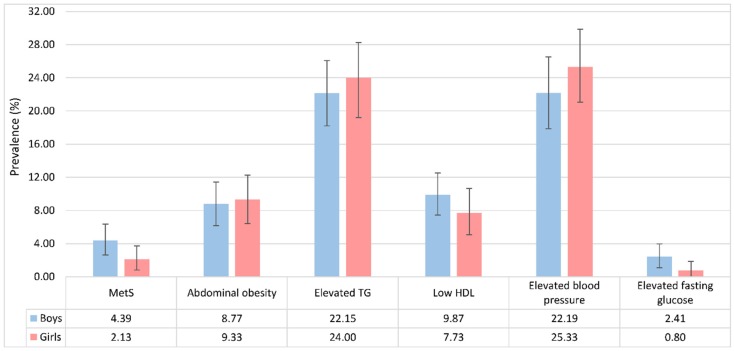
Gender-specific prevalence of paediatric metabolic syndrome (MetS) and its components. HDL: high-density lipoprotein cholesterol; TG: triglyceride.

A total of 28 children were classified as having MetS according to the NCEP-ATP III definition, which yielded an overall prevalence of 3.37% (95% CI: 2.17–4.57). Elevated blood pressure was the most prevalent MetS component, with a prevalence of 23.95% (95% CI: 20.94–27.08), followed by elevated TG (22.98%; 95% CI: 19.98–25.87). The prevalence of abdominal obesity and low HDL was similar (9.03% vs. 8.90%), while the prevalence of elevated fasting glucose was lowest (1.68%; 95% CI: 0.84–2.64). The gender-specific prevalence of MetS and its component is shown in Figure 1. The prevalence of MetS was 4.39% (95% CI: 2.63–6.36) in boys, and 2.13% (95% CI: 0.80–3.73) in girls. The prevalence estimates of elevated blood pressure, elevated TG, and abdominal obesity were higher in girls than in boys, whereas the prevalence estimates of low HDL and elevated fasting glucose were lower in girls.

**Figure 2:**
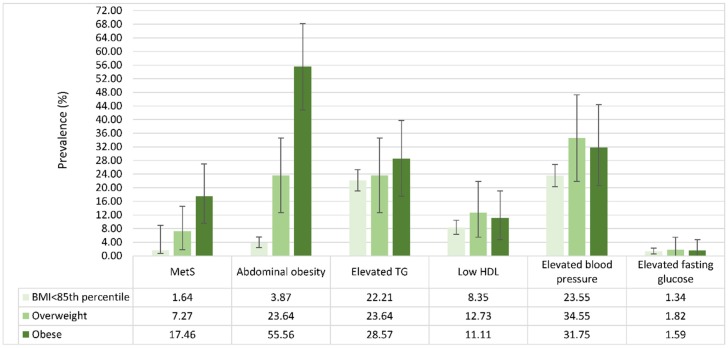
Prevalence of paediatric MetS and its components by body mass index (BMI) category.

The prevalence of MetS and its components also varied in different BMI categories (Figure 2). The prevalence of MetS was highest in obese children (17.46%; 95% CI: 7.94–9.52), and lowest in children with BMI <85th percentile (1.64%; 95% CI: 0.89–7.27). The prevalence estimates of abdominal obesity and elevated TG were both highest in obese children than in overweight children and children with BMI <85th percentile, whereas the prevalence estimates of elevated blood pressure, low HDL, and elevated fasting glucose were all highest in overweight children.

### 3.3. Correlates of MetS

After adjustment for all other variables, the results of logistic regression models (Table 3) revealed that increased BMI, hyperuricemia, and IR were all associated with the presence of MetS.

### 3.4. Agreement for Different Criteria of MetS

In children aged 10–18 years, the prevalence of MetS based on the NCEP-ATP III criteria was 3.59% (95% CI: 2.05–5.13), whereas the prevalence of MetS based on the IDF criteria was 1.37% (95% CI: 0.51–2.39). Moderate agreement (κ = 0.54) was found between these two criteria in diagnosing MetS in Chinese children (Table 4). All of the MetS cases diagnosed by the IDF criteria were also identified by the NCEP-ATP III definition.

## 4. Discussion

For the largest developing country (China), the nutritional transition in the 21st century has brought numerous health problems in children [35]. The importance of screening MetS and its components in the paediatric population has been well-demonstrated in earlier studies [6,8,26]. In our study, we reported the prevalence of MetS and its components in Chinese children using the widely adopted NCEP-ATP III criteria; to the best of our knowledge, this study is the first population-based study to examine the prevalence and distribution of MetS in a nationally representative sample of Chinese children. 

At the population level, the MetS prevalence was estimated to be between 1.8% and 2.6% in Chinese children [19]; the variation came mainly from the specific characteristics of the investigated population and the adopted MetS criteria. A meta-analysis of the MetS prevalence in Chinese children revealed that the prevalence of MetS diagnosed by NCEP-ATP III criteria increased from 2.3% in 2004–2010 to 3.2% in 2011–2014 [19]. The dramatic increasing trend may be associated with the epidemic of obesity in China [18]. Compared with the synthesised prevalence of MetS, our nation-wide study conducted in 2009 presented a slightly higher prevalence of 3.37%. Although the overall prevalence of paediatric MetS was lower than that in developed countries [26,36], the huge population in China still suggests that more than 11 million children in China were affected by this syndrome.

The results of this study suggest that the prevalence of MetS was higher in boys than in girls. However, the gender effect was not verified in the logistic regression analysis when controlled by other variables. The lack of significant gender difference in paediatric MetS is consistent with previous studies in Korean children [36], but contrasts findings in earlier Chinese studies [19]. Future studies are still needed to explore the gender-based differences in the Chinese paediatric population. The prevalence of MetS increased directly with the degrees of general obesity as assessed by BMI; the association was also confirmed by the logistic regression analysis. This finding underscores the deleterious effect of obesity in children [37]. Furthermore, hyperuricemia and IR were both found as strong risk correlates of paediatric MetS. The positive association between UA and the presence of MetS was in line with the findings of a nationally representative sample of US children and adolescents [26]; the potential coordinator may be insulin, which has a negative effect on the renal clearance of urate [26]. In both adults and children, IR has been proven to accelerate the processes underlying MetS [38]; in our study, the potential pathophysiological mechanisms of IR and MetS in the paediatric population has also been supported by the results of logistic regression analysis with adjustments of other factors.

Another aim of this study was to compare the application of NCEP-ATP III and IDF criteria in Chinese children. The Kappa statistics suggested a moderate agreement between these two criteria, and the NCEP-ATP III criteria was more sensitive than the IDF criteria. The inconsistency may mainly come from the difference of the component criteria. However, neither of the two criteria was based on the Chinese paediatric population; although ethnic-specific percentiles of WC and BP were adopted in our analysis, the cut-offs of glucose, TG, and HDL may still inappropriate. Further studies are required to determine the suitability of these cut-offs for defining elevated fasting glucose, elevated TG, and low HDL in Chinese children.

The strengths of this study include a large representative sample of the Chinese paediatric population, the use of standardised protocols and data collection procedures, the training of data collectors, as well as quality control assurance. All blood samples were analysed in a central laboratory in the capital according to clinic laboratory standards, and thus all of the abovementioned factors can largely avoid measurement bias. In addition, although the cross-sectional survey design does not allow causal conclusions between many of the correlates and the cluster components of MetS, this study still serves as the most in-depth exploration of the correlates of paediatric MetS at the national population level. 

Our study should also be interpreted in light of its limitations. Firstly, despite our efforts to define IR by taking both pubertal stages and age into consideration, the lack of an individual assessment of physical development may bring bias in the classification of IR. Secondly, we could not include all potential correlates for analysis because of the availability of data. 

## 5. Conclusions

To conclude, our study revealed the prevalence of MetS in Chinese children at the national level, and explored the correlates. Our findings indicate that the epidemic of paediatric MetS is associated with the increasing degree of general obesity as assessed by BMI, hyperuricemia, and insulin resistance. Further large-scale studies are still needed to identify appropriate criteria of MetS in the general paediatric population in China.

## Figures and Tables

**Table 1:** Insulin resistance (IR) thresholds assessed using the homeostasis model assessment (HOMA) index by pubertal status.

Stage	75th Percentile of the HOMA Index	
	Boys	Girls
Prepubertal	2.94	2.62
Pubertal	4.43	4.56
Postpubertal	4.66	3.95

**Table 2:** Basic characteristics of the study subjects by presence of metabolic syndrome (MetS) and its components using the National Cholesterol Education Program Adult Treatment Panel III (NCEP-ATP III) criteria.

Characteristic	Total (*n* = 831)	With MetS (*n* = 28)	With Abdominal Obesity (*n* = 75)	With Elevated TG (*n* = 191)	With Low HDL (*n* = 74)	With Elevated Blood Pressure (*n* = 199)	With Elevated Fasting Glucose (*n* = 14)
Gender							
Boys (%)	456 (54.87%)	20 (71.43%)	40 (53.33%)	101 (52.88%)	45 (60.81%)	104 (52.26%)	11 (21.43%)
Girls (%)	375 (45.13%)	8 (28.57%)	35 (46.67%)	90 (47.12%)	29 (39.19%)	95 (47.74%)	3 (78.57%)
Residence							
Urban (%)	347 (42.68%)	11 (39.29%)	31 (41.33%)	84 (45.41%)	28 (38.89%)	77 (38.69%)	8 (61.54%)
Rural (%)	466 (57.32%)	17 (60.71%)	44 (58.67%)	101 (54.59%)	44 (61.11%)	122 (61.31%)	5 (38.46%)
Age (years)	12.39 ± 3.05	11.83 ± 2.59	11.67 ± 2.52 ^$^	12.96 ± 2.88 ^€^	13.09 ± 2.86 ^£^	11.76 ± 3.00 ^§^	12.87 ± 3.45
Weight (kg)	39.64 ± 13.13	47.47 ± 14.93 *	48.43 ± 15.11 ^$^	41.97 ± 12.79 ^€^	42.55 ± 14.61	39.53 ± 14.08	39.48 ± 11.15
Height (cm)	147.3 ± 15.85	150.31 ± 11.08	150.34 ± 12.94 ^$^	149.81 ± 13.77 ^€^	150.23 ± 16.02	145.27 ± 16.27 ^§^	148.85 ± 17.57
BMI (kg/m^2^)	17.77 ± 3.33	20.73 ± 5.02 *	21.04 ± 4.48 ^$^	18.32 ± 3.60 ^€^	18.30 ± 4.03	18.12 ± 3.51	17.57 ± 2.88
WC (cm)	63.26 ± 9.61	78.90 ± 10.52 *	79.30 ± 7.77 ^$^	65.51 ± 10.06 ^€^	66.26 ± 11.98 ^£^	63.74 ± 11.30	64.16 ± 7.18
SBP (mmHg)	100.04 ± 13.03	111.04 ± 17.35 *	104.38 ± 16.38 ^$^	101.64 ± 13.83	99.97 ± 13.39	111.95 ± 10.63 ^§^	99.88 ± 14.52
DBP (mmHg)	66.67 ± 9.50	76.59 ± 11.48 *	69.71 ± 10.59 ^$^	68.00 ± 10.79	66.76 ± 10.06	76.05 ± 7.54 ^§^	65.67 ± 9.43
Hb (g/L)	137.65 ± 16.54	137.04 ± 11.29	136.05 ± 12.13	139.51 ± 17.34	140.36 ± 19.57	137.59 ± 15.99	145.21 ± 12.14
UA (μmol/L)	310.14 ± 84.99	390.18 ± 74.15 *	339.96 ± 85.15 ^$^	345.45 ± 89.49 ^€^	350.84 ± 92.60 ^£^	308.70 ± 85.76	384.29 ± 92.03 ^¥^
TC (mmol/L)	3.88 ± 0.70	4.10 ± 0.82	3.99 ± 0.76	4.12 ± 0.82 ^€^	3.51 ± 0.62 ^£^	3.92 ± 0.68	4.61 ± 1.31
HDL (mmol/L)	1.44 ± 0.53	1.13 ± 0.42 *	1.49 ± 1.28	1.29 ± 0.37 ^€^	0.92 ± 0.09 ^£^	1.42 ± 0.32	1.31 ± 0.33
LDL (mmol/L)	2.21 ± 0.88	2.18 ± 0.66	2.27 ± 0.59	2.21 ± 0.77	2.01 ± 0.57	2.18 ± 0.60	2.60 ± 1.41
TG (mmol/L)	1.01 ± 0.72	2.46 ± 1.35 *	1.25 ± 0.84 ^$^	1.94 ± 0.95^€^	1.57 ± 1.20 ^£^	1.07 ± 0.66	2.42 ± 2.22 ^¥^
Glucose (mmol/L)	4.89 ± 0.80	5.43 ± 1.65	4.99 ± 0.97	5.15 ± 1.33 ^€^	4.79 ± 0.75	4.89 ± 0.53	8.59 ± 3.42 ^¥^
Insulin (μU/mL)	11.25 (8.08–16.68)	23.71 (16.17–34.76) *	18.59 (13.26–27.53) ^$^	13.65 (9.83–23.45) ^€^	12.27 (7.50–16.91)	12.37 (8.91–18.33) ^§^	27.01 (17.53–42.79) ^¥^
HOMA-IR	2.40 (1.71–3.66)	4.94 (3.08–7.69) *	3.86 (2.71–6.30) ^$^	2.97 (2.15–5.33) ^€^	2.48 (1.51–3.66)	2.70 (1.95–4.10) ^§^	9.26 (5.84–11.53) ^¥^

Note: data are *n* (%), means ± SD, median with lower and upper quartiles (for insulin and homeostasis model assessment insulin resistance, HOMA-IR). * Significantly different from subjects without MetS (*p* < 0.05); ^$^ Significantly different from subjects without abdominal obesity (*p* < 0.05); ^€^ Significantly different from subjects without elevated TG (*p* < 0.05); ^£^ Significantly different from subjects without low HDL (*p* < 0.05); ^§^ Significantly different from subjects without elevated blood pressure (*p* < 0.05); ^¥^ Significantly different from subjects without elevated fasting glucose (*p* < 0.05). BMI: body mass index; WC: waist circumference; BP: blood pressure; SBP: systolic blood pressure; DBP: diastolic blood pressure; Hb: haemoglobin; UA: uric acid; TC: total cholesterol; HDL: high-density lipoprotein cholesterol; LDL: low-density lipoprotein cholesterol; TG: triglyceride.

**Table 3:** Logistic regression analysis of the correlates associated with paediatric MetS.

	Adjusted OR (95% CI)	*p* Value
BMI category		
<85th percentile ^r^	1.00	
Overweight	3.68 (1.09–12.50)	0.036
Obesity	7.33 (2.84–18.90)	<0.001
Hyperuricemia		
No ^r^	1.00	
Yes	4.66 (1.93–11.25)	0.001
IR		
No ^r^	1.00	
Yes	3.11 (1.31–7.41)	0.010

Note: ^r^ Reference category. Variables included in the adjusted model were age, gender, residence, BMI category, abdominal obesity, hypertension, anaemia, hyperuricemia, IR, and lipid disorders (elevated TC, low HDL, elevated LDL, elevated TG, and dyslipidaemia).

**Table 4:** Agreement between the NCEP-ATP III and International Diabetes Federation (IDF) criteria in diagnosing MetS.

		MetS Diagnosed by NCEP-ATP III			κ (95% CI)
		Total	+	-	
MetS diagnosed by IDF	Total	585	21	564	0.54 (0.30–0.74)
	+	8	8	0	
	−	577	13	564	

Note: + with MetS; − without MetS.
